# Spatial Variation and Controlling Factors of H and O Isotopes in Lancang River Water, Southwest China

**DOI:** 10.3390/ijerph16244932

**Published:** 2019-12-05

**Authors:** Kunhua Yang, Guilin Han, Jie Zeng, Bin Liang, Rui Qu, Jinke Liu, Man Liu

**Affiliations:** Institute of Earth Sciences, China University of Geosciences, Beijing 100083, China; kunhuayang@cugb.edu.cn (K.Y.); zengjie@cugb.edu.cn (J.Z.); liangbin@cugb.edu.cn (B.L.); qurui@cugb.edu.cn (R.Q.); liujinke@cugb.edu.cn (J.L.); lman@cugb.edu.cn (M.L.)

**Keywords:** hydrogen isotope, oxygen isotope, groundwater, glacier, dams, water cycle, Lancang River, Qinghai–Tibetan Plateau

## Abstract

Climate changes and other human activities have substantially altered the hydrological cycle with respect to elevation. In this study, longitudinal patterns in the stable isotopic composition (δ^2^H and δ^18^O) of Lancang River water, originating from the Qinghai–Tibetan Plateau, are presented, and several controlling factors in the wet season are hypothesized. Lancang River water δ^2^H (−145.2‰ to −60.7‰) and δ^18^O (−18.51‰ to −8.49‰) were low but close to those of the Global Meteoric Water Line. In the upper reaches of the river, δ^2^H decreased longitudinally, potentially due to groundwater inputs and melting ground ice in the headwater zone and to an increasing proportion of glacier meltwater with decreasing elevation. In the middle reaches of the river, δ^2^H values increased slowly moving downstream, likely due to shifts in precipitation inputs, as evidenced by the isotopic composition of tributaries to the main stream. In the lower reaches of the river, the isotopic composition was relatively invariant, potentially related to the presence of large artificial reservoirs that increase the water resident time. The results reveal different hydrological patterns along an alpine river in central Asia associated with both natural and anthropogenic processes. Understanding the degree and type of human interference with the water cycle in this region could improve water management and water security.

## 1. Introduction

Climate changes and human activities have impacted the hydrological cycles and local environment in the Qinghai–Tibetan Plateau, which is the largest and highest plateau in the world and is called the “Water Tower of Asia” and the “Third Pole” [1,2,3]. The Lancang-Mekong river originates from the Qinghai–Tibetan Plateau and is one of ten largest Asian rivers [4]. Covering 24% of the total area of the Lancang-Mekong River basin, the Lancang River basin (LRB) has recorded the annual discharge of 61.7 km^3^ of water at the Jinghong station, which accounts for about 13.5% of the total annual discharge to the Lower Mekong River [5]. Under the impacts of natural and anthropogenic activities, the water cycle and distribution of water resources have been altered in the LRB [6,7,8]. In mountainous areas, global climate change is related to catchment weathering, mountain glacier shrinkage, natural hazards, and watershed hydrology [5,9,10,11,12]. Glaciers represent a local water resource in the mountains and release substantial melt water in the summer and early autumn [13]. The literature shows that the glaciers in the LRB have a comparable area loss rate compared to that of several high Asian mountain regions, and the higher rates in the LRB have been found in the Meili Snow Mountain range, west of the Deqin county [5]. The melting of glaciers have explicitly impacted the runoff variations of the Lancang River [14]. On the other hand, human activities have factually exerted an influence on watershed hydrology [15,16]. For instance, cascade dams construction has significantly influenced the hydrology, landscape, riparian functions, and carbon cycle of rivers. [3,17,18]. Owing to its particularly high mean elevation, the LRB has a considerable hydropower potential and suitability to develop hydropower; more than 50 commissioned dams are essential for providing electricity, supporting irrigation, and supplying water [19,20].

The stable hydrogen and oxygen isotopes have been widely used to trace the impacts of climate changes and other human activities on the hydrological cycles in watersheds. In the 2000s, an isotopic database named the Global Network of Isotopes in Rivers (GNIR) was launched by the International Atomic Energy Agency (IAEA) to collect H and O isotopic data from about 20 large rivers of the world [21]. Moreover, the isoscapes (i.e., the spatiotemporal isotope distributions) of river water are usually connected with those of precipitation, which are influenced by altitude, latitude, temperature, and precipitation amount [22]. The linear distribution of δ^2^H and δ^18^O in river water along the Global Meteoric Water Line (GMWL) is considered as evidence of the meteoric origin [23]. Consequently, the isotopic composition of river water can be associated with precipitation, temperature, altitude, and snow/glacier melting [24,25,26]. Also, the interaction between groundwater and surface water can be traced by H and O isotopes [27,28]. Further, stable H and O isotopes have demonstrated the influence of anthropogenic processes upon the water cycle [29,30]. Cascade dams are massively constructed on the main stream and major tributaries of the Lancang-Mekong River, which may be accelerating and magnifying the long-term effect of climate changes and global warming [31]. The spatio-temporal variation of H and O isotopes reflects the effects of trapping the river and of water regulation in some rivers [32,33]. Regarding the damming effect, the seasonal distribution of stable oxygen isotopes in the Changjiang River water shows a time lag in responding to meteoric precipitation [34], and cascade dams increase the resident time of river water and change the isotopes of surface water, bottom water, and released water in the reservoirs, which indeed shows their influence on the water cycle [35].

To investigate the impacts of climate changes and human activities on hydrological processes, the longitudinal variation of stable H and O isotopes in Lancang River water was discussed, focusing in particular on: (1) the isotopic composition of Lancang River water in comparison with those of other Asian rivers; (2) the spatial variation of isotopes with geospatial variables in the upper reaches, middle reaches, and lower reaches; (3) the main factors controlling the isotopes’ distribution in different reaches. This study examined the isotopic data of the Lancang River water from mouth to source in the main stream and tributaries to provide a scientific viewpoint on the influence of natural and anthropogenic processes upon the water cycle in mountainous areas. The results can promote further research on eco-hydrology and contribute to the water management and water security in the Lancang River basin.

## 2. Materials and Methods 

### 2.1. Background of the Study Area

**Figure 1:**
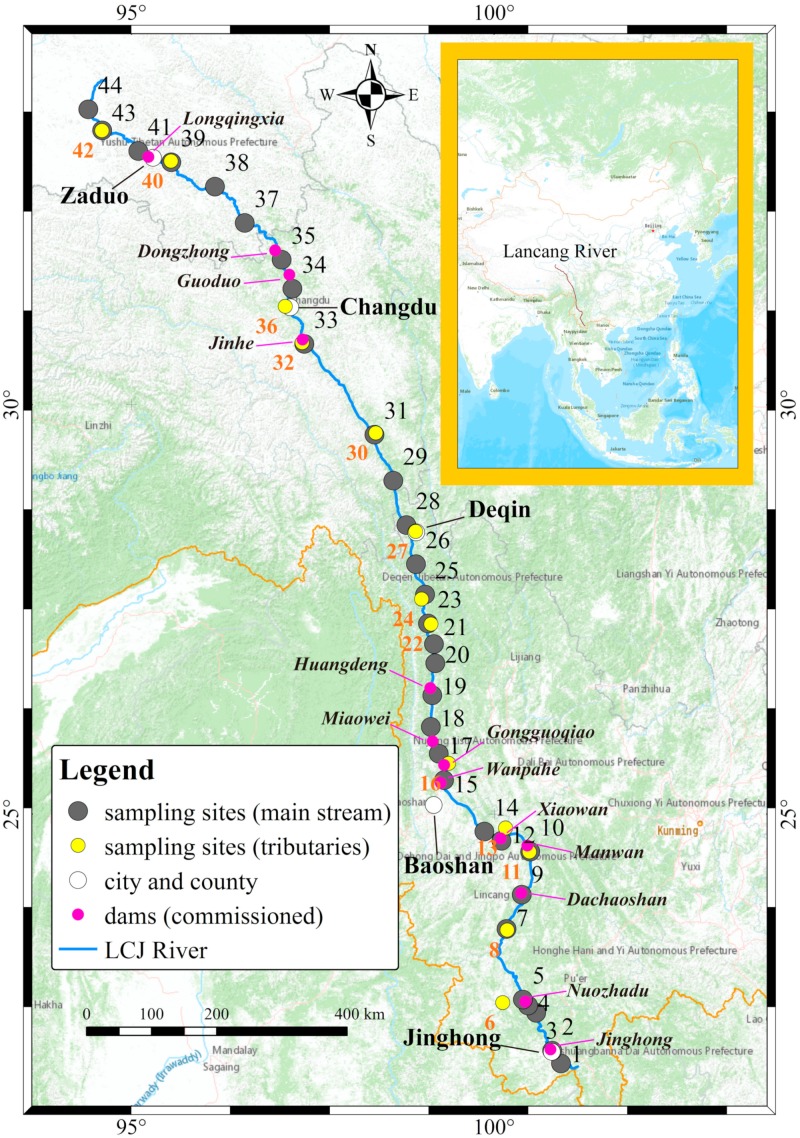
Location of the Lancang River and sampling sites and distribution of commissioned dams along the main channel.

The Lancang River is situated in southwest China, central Asia (94°~102° E, 21°~34° N), and the length of its main channel is 2161 km (Figure 1). It originates from northwest Zaduo County, Qinghai Province, and zigzags through the Qinghai–Tibetan Plateau, the Hengduan Mountains, and the Yunnan–Guizhou Plateau. The Lancang River is the upper reaches of the Lancang-Mekong River, and many dams were constructed along its main channel (Figure 1) and some tributaries [20]. With an elevation from circa (ca.) 6000 m to 500 m, the climatic zones in the Lancang River catchment include a Frigid Zone, a Frigid–Temperate Zone, a Temperate Zone, a Sub-Tropical Zone, and a Tropical Zone [36]. Affected by the Asian monsoon systems, the LRB receives abundant precipitation in the summer. During the wet season (May to October), precipitation in the LBR varies with the geospatial variables [37]. Nevertheless, the annual precipitation generally increases from the headstream moving downstream, from 299 mm to 4925  mm, along the latitudinal gradient [37]. According to the climate, the geography, and the hydrology, the Lancang River basin can be divided into three sections, including a permafrost region (28°30′~34° N), the Hengduan region (26°~28°30′ N), and the Yunnan Region (21°~26° N).

### 2.2. Sampling Processes

In total, 44 river water samples were collected in the Lancang River catchment from mouth to source on 18 July–7 August 2019 (Figure 1). The water was generally taken from the river bank or the middle of the channel from bridges at a sampling depth of ca. 50 cm. In each sampling site, water temperature, dissolved oxygen, and total dissolved solids (TDS) were determined by a YSI multi-parameter meter (Pro Plus, YSI Inc. /Xylem Inc., Ohio, USA), and longitude, latitude, and altitude were recorded by GPS. The water samples were filtered through 0.22 μm cellulose acetate membranes (Yibo Factory, Haining, China) and sealed in pre-cleaned PET (polyethylene terephthalate) bottles using Parafilm ® M film (Bemis Company Inc., Wis., USA).

### 2.3. Isotope Analysis

The stable isotopic composition (δ^2^H and δ^18^O) was analyzed with laser spectroscopy techniques at the Institute of Geographic Sciences and Natural Resources Research, Chinese Academy of Sciences, using a Triple-Isotopic Water Analyzer (Model TIWA-45-EP, Los Gatos Research Inc., USA). For monitoring the data, one isotopic standard was inserted every three samples. Every sample/standard was analyzed six times, discarding the first two measurements to avoid the memory effect. Then, the last four measurements were averaged, providing the final value for the sample or standard. The measurement results of δ^2^H and δ^18^O were expressed as:

δ^2^H (‰) = [(^2^H/^1^H)*_sample_*/(^2^H/^1^H)*_standard_* − 1] × 10^3^ (1)

δ^18^O (‰) = [(^18^O/^16^O)*_sample_*/(^18^O/^16^O)*_standard_* − 1] × 10^3^ (2)

The isotope data were reported in per mill (‰) relative to the Vienna Standard Mean Ocean Water (V-SMOW), and the measurement precision was ±0.5‰ (1σ) for δ^2^H and ±0.1‰ (1σ) for δ^18^O.

## 3. Results and Discussion

### 3.1. H and O Isotopic Composition

For Lancang River water, the δ^2^H values ranged from −145.2‰ to −60.7‰, and the mean value was −98.4‰, whereas the δ^18^O values ranged from −18.51‰ to −8.49‰, and the mean value was −13.55‰ (Table 1). Equation (3) showed a significant linear correlation between δ^2^H and δ^18^O:

δ^2^H = 7.72 × δ^18^O + 6.31 (*n* = 44, r^2^ = 0.97, *p* < 0.01) (3)

**Figure 2:**
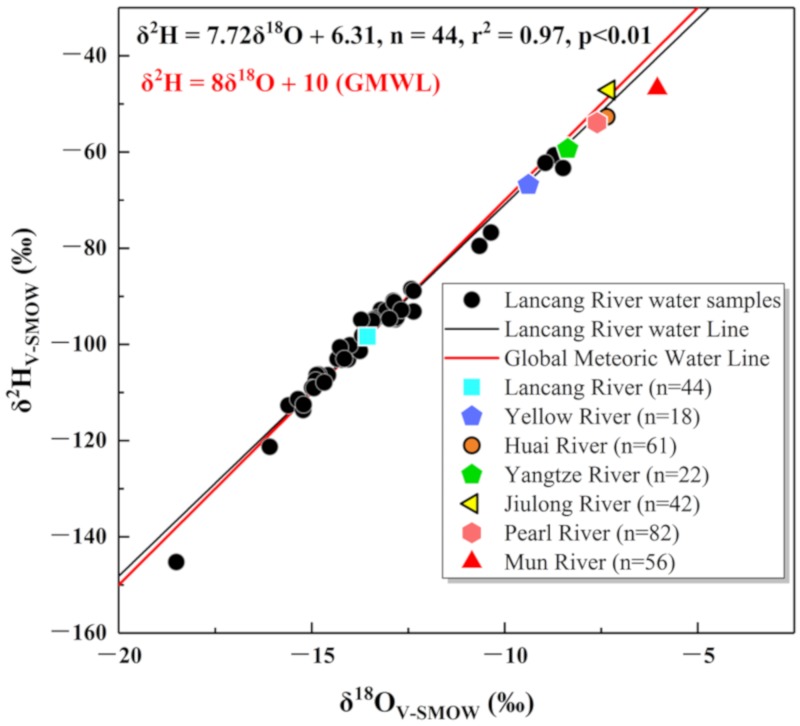
Scatter plots of δ^18^O and δ^2^H in the wet season in the river water of the Lancang River (this study), the Yellow River [38], the Huai River [30,39], the Yangtze River [40], the Jiulong River [29], the Pearl River [41], and the Mun River [42].

Figure 2 shows that the Lancang River Line was almost coincident with the GMWL, and most of the Lancang River water samples were generally located on the GMWL. Hence, Lancang River water did not experience significant evaporation.

We compared the stable isotopic composition in the wet season of the water of seven rivers in Asia, characterized by different climate types and altitudes, including the Lancang River (this study), the Yellow River, mainly in the temperate zone [38], the Huai River in the temperate–subtropical transition zone [30,39], the Yangtze River, mainly in the subtropical zone [40], the Jiulong River in the subtropical zone [29], the Pearl River in the subtropical zone [41], and the Mun River in the tropical zone [42]. In Figure 2, the mean values of the isotopic composition of different rivers were obviously different, especially when comparing the Lancang River with other rivers. The isotopic data for the Lancang River showed the lowest values. Over all, δ^2^H and δ^18^O in different rivers showed an increasing trend with decreasing latitude, except for the Jiulong River, which is close to the ocean, and the Huai River, which evaporates significantly. Therefore, the low isotopic composition of Lancang River water was most likely related to its frigid and frigid–temperate climate.

### 3.2. Spatial Distribution of Isotopes along the Flow Path

**Figure 3:**
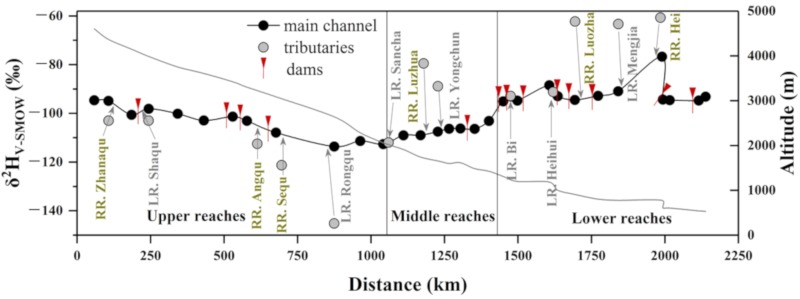
Hydrogen isotopic composition of river water along the flow path. LR represents the left bank of the river, and RR represents the right bank of the river.

The spatial distribution of δ^2^H along the Lancang River flow path is displayed in Figure 3. In general, the δ^2^H values decreased rapidly along the flow path in the upper reaches and then increased slowly in the middle reaches. The δ^2^H values were almost homogenous in the lower reaches, except for sample No. 4, which was likely influenced by the Hei River water on the right bank. On the other hand, the differences in stable isotopic composition of river water between the left branches and the right branches were not significant.

**Figure 4:**
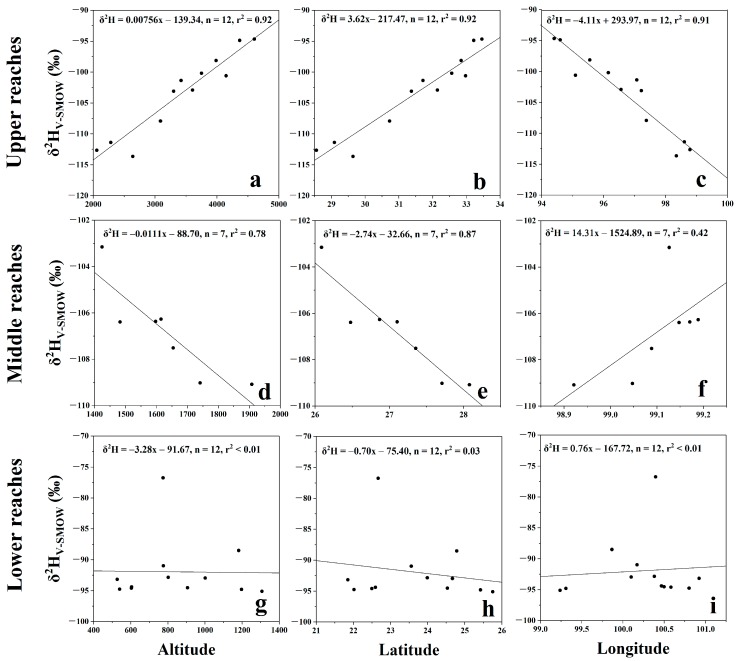
Differences among three reaches in the relationship between δ^2^H of river water in the main channel and altitude/latitude/longitude. (**a**) Positive correlation between δ^2^H and altitude in the upper reaches. (**b**) Positive correlation between δ^2^H and latitude in the upper reaches. (**c**) Negative correlation between δ^2^H and longitude in the upper reaches. (**d**) Negative correlation between δ^2^H and altitude in the middle reaches. (**e**) Negative correlation between δ^2^H and latitude in the middle reaches. (**f**) Positive correlation between δ^2^H and longitude in the middle reaches. (**g**) No obvious linear correlation between δ^2^H and altitude in the lower reaches. (**h**) No obvious linear correlation between δ^2^H and latitude in the lower reaches. (**i**) No obvious linear correlation between δ^2^H and longitude in the lower reaches.

The δ^2^H of river water showed obvious variability. In the upper reaches, δ^2^H values in the main channel increased linearly with increasing altitude (Figure 4a) and latitude (Figure 4b), while δ^2^H decreased with increasing longitude (Figure 4c). However, we observed an opposite linear relationship between δ^2^H and altitude/latitude/longitude in the main channel of the middle reaches with respect to the upper reaches (Figure 4d–f). In the lower reaches, we did not observe a linear correlation between δ^2^H and altitude/latitude/longitude (Figure 4g–i). In the mountain area, a negative linear relationship between the changes of δ^2^H in relation to precipitation and elevation indicated the “altitude effect”, which appears when river water is mainly recharged by rainwater [29]. For Lancang River water, the δ^2^H and altitude showed a negative correlation in the middle channel and a positive correlation in the upper channel. In addition, the relationship between latitude/longitude and δ^2^H can be explained by the northwest-to-southeast flow direction and elevation decrease. Therefore, it can be inferred that rainfall is the main controlling factor of river water δ^2^H in the middle reaches but not in the upper reaches. On the other hand, we consider that the river water in the main channel of the lower reaches is most likely affected by anthropogenic activities, such as river impounding, owing to no obvious linear relationship between δ^2^H and altitude [29].

### 3.3. Inputs of Groundwater, Ground Ice, and Glaciers Meltwater in the Permafrost Region

The presence of stable isotopes in run-off is connected with that in recent rainfall as a result of direct recharging from rainwater [29]. For example, in northwestern Tibetan Plateau, the stable isotopic composition of precipitation in the summer shows an increasing trend with decreasing elevation, i.e., the “altitude effect” [43]. Regarding the Lancang River, its water in the middle reaches is significantly recharged by local precipitation, as shown by the negative correlation between δ^2^H and altitude (Figure 4d). 

Previous studies suggested that monsoon precipitation is the main factor sustaining the river flow up to Changdu City [44,45]. However, Figure 4a reflects the positive correlation between δ^2^H in Lancang River water and elevation in the upper reaches, which is in contrast with what observed for those rivers mainly recharged by precipitation [29]. In the upper reaches of the Lancang River (altitude >2000 m), the permafrost regions or glaciers are widely distributed [46]. In the central Qinghai–Tibet Plateau, δ^2^H of the active layer of water and of ground ice ranged from −130.2‰ to −64.2‰ and was lower than that of local precipitation [47]. On the North Slope of Alaska, δ^18^O of ground ice is about −24.4‰ [48]. In a word, stable isotopic value is very low in ground ice, which generally decrease as soil depth increases. In the northeastern Qinghai–Tibet Plateau, in June–August, the δ^18^O content of glacier snowmelt water (from −9.51‰ to −11.69‰) was lower than those of stream water, rainwater, and baseflow, and the baseflow had the highest TDS content (ca. 402 mg/L) [49]. Therefore, we tried to estimate the inputs of groundwater and glacier meltwater on river water using stable isotopes.

**Figure 5:**
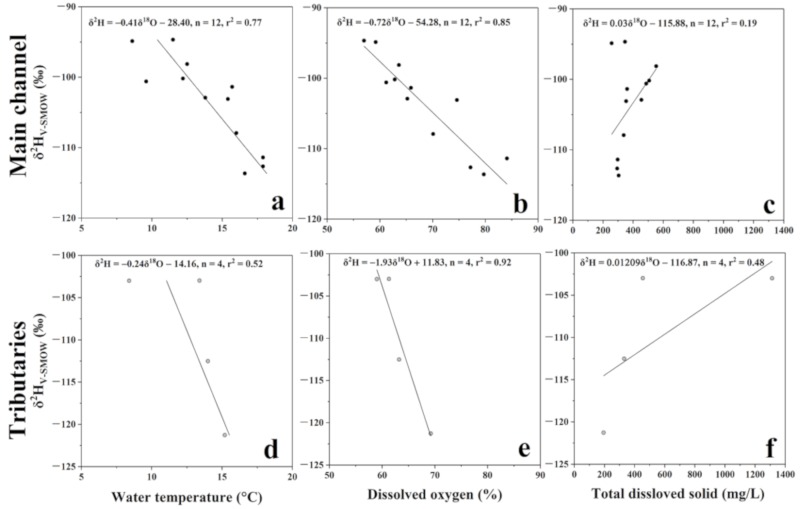
Relationship between δ^2^H and water temperature/dissolved oxygen/total dissolved solid of river water in the main channel and tributaries in the upper reaches. (**a**) Cross plot of δ^2^H and water temperature of the river water in the main channel. (**b**) Cross plot of δ^2^H and dissolved oxygen in the river water in the main channel. (**c**) Cross plot of δ^2^H and total dissolved solid of the river water in the main channel. (**d**) Cross plot of δ^2^H and water temperature of the river water in the tributaries. (**e**) Cross plot of δ^2^H and dissolved oxygen of the river water in the tributaries. (**f**) Cross plot of δ^2^H and total dissolved solid of the river water in the tributaries.

In the troposphere, air temperature decreases with increasing elevation. In the upper reaches of the Lancang River, water temperature and dissolved oxygen showed a similar trend to that of altitude (Figure 5a–d). It means that glaciers and ground ice in the permafrost zone melted more longitudinally. Moreover, precipitation or meltwater infiltrated into the active layer that may interact with surface water via lateral flow or overland flow [49,50]. In the upstream before the Zaduo County, the mean δ^2^H value of river water was −98.3‰, and the mean TDS value was 600 mg/L. Thus, it can be inferred that the upstream river is mainly recharged by ground ice water from the active layer. With elevation decrease, the TDS values and δ^2^H values of tributaries’ water decreased rapidly, (Figure 5e,f). This suggests that the headwater zone of the Lancang River was fed by ground ice water, groundwater, and the precipitation, and the contribution of glaciers meltwater increased longitudinally. Therefore, the isotopic composition of river water in the main channel decreased along the flow path, owing to the recharge from tributaries water which were more enriched in light isotopes as the elevation decreased. On the other hand, Figure 5c,f show that the TDS values of the Lancang River water is similar to those of rivers in a low-elevation area, suggesting that the chemical denudation rate in the alpine arid area can be comparable to the world average value [51].

### 3.4. Impacts of Cascade Dams on Hydrology

In the flood season, the amount of abandoned water increases due to the increase of the reservoir inflow, which augments the hydrological alteration between upstream and downstream areas [3]. In the lower reaches of the Lancang River, the content of stable isotopes of the river water was relatively invariant in the main channel (Figure 3) and did not show an obvious linear relationship with altitude/latitude/longitude (Figure 4g–i). Intensive large artificial reservoirs are present in the lower reaches, such as the Gongguoqiao, Xiaowan, and Nuozhadu hydropower stations (Figure 1). Hence, the effect of damming should be condisered as the cause of this phenomenon. The damming effect consists of the trapping effect and water regulation of reservoirs, especially in rivers with cascade dams. In many studies, the homogenous content of isotopes in river water is considered the results of cascade reservoirs [29,42]. Besides the isotopic values, water temperature and total dissolved solids in the river water were also invariant in the main channel of the lower reaches, which supports the presence of the damming effect. However, the δ^2^H value of the sample LCJ05 was high and unordinary, which could be caused by the sampling site being too close to the Hei River.

## 4. Conclusions

We examined the spatial characteristics of isotopes in the Lancang River and several controlling factors with respect to the elevation in the wet season. The results showed that the stable isotopic composition (δ^2^H and δ^18^O) of the Lancang River water was lower than that of the Yellow River, the Huai River, the Yangtze River, the Jiulong River, and the Mun River in Asia. The Lancang River Water Line was calculated as δ^2^H = 7.72 × δ^18^O + 6.31 (*n* = 44, r^2^ = 0.97) and resulted very close to the GMWL. Isotopic values differed in different reaches, for which the longitudinal trend of δ^2^H was different. In the upper reaches, the river water appeared to be mainly recharged by glaciers meltwater, which was enriched in lighter stable isotopes and whose contribution was greater in low-elevation areas, although the most upstream zone was also affected by ground ice water. In the middle reaches, groundwater and glacier water recharging was not obvious, and the water appeared to be mainly recharged by precipitation, as shown by the generally increasing isotopic values with decreasing elevation. In the lower reaches, the δ^2^H values were relatively invariant, suggesting a significant damming effect. Therefore, stable isotopes demonstrated the presence of different hydrological processes in the Lancang River catchment, including the interaction between river water and groundwater, glacier meltwater and precipitation, and the impact of anthropogenic activities (damming) on the water cycle.

## Figures and Tables

**Table 1:** Locations, water temperature (T), dissolved oxygen (DO), total dissolved solid (TDS), and isotope composition of river water samples.

Reaches	Section	Samples	Latitude	Longitude	Altitude	T	DO	TDS	δ^2^H	δ^18^O
			° N	° E	m	℃	%	mg/L	‰	‰
upper reaches	Main channel	LCJ44	33.46	94.40	4604	11.5	57.0	346.5	−12.99	−94.7
		LCJ43	33.22	94.60	4368	8.6	59.2	257.4	−13.72	−94.9
		LCJ41	32.98	95.09	4148	9.6	61.2	487.5	−14.27	−100.6
		LCJ39	32.84	95.55	3986	12.5	63.6	552.5	−13.69	−98.1
		LCJ38	32.56	96.15	3751	12.2	62.8	507.0	−14.01	−100.2
		LCJ37	32.14	96.56	3606	13.8	65.2	455.0	−14.29	−102.9
		LCJ35	31.71	97.07	3420	15.7	65.9	360.1	−13.75	−101.4
		LCJ34	31.37	97.22	3300	15.4	74.6	353.6	−14.06	−103.1
		LCJ33	30.72	97.38	3087	16.0	70.1	337.4	−14.67	−107.9
		LCJ31	29.64	98.35	2639	16.6	79.7	304.2	−15.22	−113.7
		LCJ29	29.09	98.61	2284	17.9	84.1	297.7	−15.35	−111.4
		LCJ28	28.55	98.79	2058	17.9	77.2	293.8	−15.60	−112.7
	Tributaries	LCJ42	33.21	94.59	4382	8.4	59.0	1313.0	−14.15	−103.0
		LCJ40	32.86	95.55	3980	13.4	61.3	455.0	−14.33	−103.0
		LCJ36	31.16	97.12	3243	14.0	63.2	330.9	−15.21	−112.5
		LCJ32	30.73	97.35	3175	15.2	69.2	194.4	−16.09	−121.3
		LCJ30	29.66	98.37	2853	16.2	72.5	204.8	−18.51	−145.2
middle reaches	Main channel	LCJ26	28.08	98.92	1908	16.9	90.1	267.8	−14.94	−109.1
		LCJ25	27.71	99.05	1741	17.2	87.3	254.8	−14.99	−109.0
		LCJ23	27.36	99.09	1654	17.3	85.1	250.3	−14.90	−107.5
		LCJ21	27.10	99.17	1597	19.7	88.8	257.4	−14.87	−106.4
		LCJ20	26.87	99.19	1615	24.5	93.9	251.6	−14.83	−106.3
		LCJ19	26.48	99.15	1482	18.1	84.5	258.1	−14.58	−106.4
		LCJ18	26.09	99.13	1424	18.4	89.0	241.8	−14.22	−103.2
	Tributaries	LCJ27	28.47	98.92	3104	12.3	72.1	137.8	−15.36	−112.0
		LCJ24	27.65	99.00	1741	15.9	84.0	52.7	−10.65	−79.5
		LCJ22	27.35	99.13	1720	19.9	79.6	120.9	−12.36	−88.9
lower reaches	Main channel	LCJ17	25.75	99.24	1307	18.4	87.3	232.1	−13.43	−95.1
		LCJ15	25.42	99.31	1197	18.8	95.1	233.4	−13.41	−94.8
		LCJ14	24.78	99.87	1181	23.2	107.0	234.0	−12.42	−88.5
		LCJ12	24.67	100.10	1001	20.0	84.6	247.7	−13.06	−93.0
		LCJ10	24.53	100.50	906	20.7	82.5	252.9	−13.34	−94.5
		LCJ9	23.99	100.38	802	21.6	79.9	245.7	−13.21	−92.8
		LCJ7	23.56	100.17	776	22.4	83.0	239.9	−12.88	−91.0
		LCJ5	22.67	100.40	774	28.4	102.2	181.4	−10.35	−76.7
		LCJ4	22.59	100.47	605	20.6	62.8	237.3	−12.80	−94.4
		LCJ3	22.50	100.58	603	20.7	59.3	241.2	−12.89	−94.6
		LCJ2	22.02	100.80	540	22.1	70.6	224.7	−12.84	−94.8
		LCJ1	21.85	100.92	527	22.2	68.7	236.0	−12.36	−93.2
	Tributaries	LCJ16	25.63	99.37	1293	22.7	83.3	202.2	−12.68	−92.9
		LCJ13	24.83	100.16	1189	24.1	96.5	238.6	−12.86	−91.2
		LCJ11	24.53	100.49	901	25.1	81.5	98.2	−8.95	−62.3
		LCJ8	23.54	100.18	776	28.6	91.0	156.0	−8.49	−63.3
		LCJ6	22.63	100.12	827	24.8	84.7	100.8	−8.71	−60.7
